# Measurements of Small Frequency Differences by Dual Mode 4 MHz Quartz Sensors

**DOI:** 10.3390/s23063220

**Published:** 2023-03-17

**Authors:** Vojko Matko

**Affiliations:** Faculty of Electrical Engineering and Computer Science, University of Maribor, Koroška c. 46, 2000 Maribor, Slovenia; vojko.matko@um.si; Tel.: +386-2-220-7111

**Keywords:** small frequency difference measurement, temperature-compensated quartz sensors, differential sensors, quartz oscillators, transitions-through-zero method

## Abstract

We proposed a method for measuring frequency differences of the order of a few Hz with an experimental error lower than 0.0001% by using two 4 MHz quartz oscillators, the frequencies of which are very close (a few 10 Hz difference) due to the dual mode operation (differential mode with two temperature-compensated signal frequencies or a mode with one signal and one reference frequency). We compared the existing methods for measuring frequency differences with the new method which is based on counting the number of transitions through zero within one beat period of the signal. The measuring procedure requires equal experimental conditions (temperature, pressure, humidity, parasitic impedances etc.) for both quartz oscillators. To ensure equal resonant conditions for oscillation two quartz crystals are needed, which form a temperature pair. The frequencies and resonant conditions of both oscillators must be almost equal, which is achieved by an external inductance or capacitance. In such a way, we minimized all the external effects and ensured highly stable oscillations and high sensitivity of the differential sensors. The counter detects one beat period by an external gate signal former. By using the method of counting transitions through zero within one beat period, we reduced the measuring error by three orders of magnitude, compared to the existing methods.

## 1. Introduction

Quartz oscillators are widely used in sensor technology because of the stability of crystal oscillation [1,2,3,4,5]. Quartz crystals have a temperature dependence of a few ppm [6,7,8,9,10] which can be significantly reduced (compensated) by using two equal oscillators and two crystals, which form a temperature pair. An even better compensation is enabled by the switching method [11]. Temperature compensation is of a special importance when the frequency difference between the oscillators is very low (a few Hz), providing that one can influence the equivalent electric model of the quartz crystals which act as a differential sensor [6,12,13,14,15]. In such a way, extremely low changes of reactance due to a change in some external (nonelectrical) physical quantity can be detected (for capacitance changes in a region of zF and inductance changes in a region of pH), which is applicable for strain sensing, nanopositioning, measurements of an eccentric motion, dielectric properties of liquids and density of liquids, bacterial adhesion, middle-ear hearing devices, visualization of hidden dielectric objects, etc. [4,16,17,18,19,20,21,22,23]. Because the quartz crystal-based sensors transform a reactance into a frequency, they are applicable in biosensing, medicine and specific measurements in chemistry [24,25,26,27,28]. If the frequencies of both quartz oscillators are shifted by only a few (10 or 100) Hz, the temperature effects are equal for both oscillators and the quartz crystals (resonators) [29], and their frequency difference is practically independent of the temperature variation in the region 0–50 °C. However, when we measure the frequency difference (the crystals in the oscillator act as sensors), the measurement error depends on the chosen method [30]. If changes of the equivalent circuit of the quartz crystals are in the aF or zF region (in the case of the capacitive effect) or in the pH region (in the case of an inductive effect), changes in the resonant frequency, which are typical in measurements of a mechanical displacement, nanopositioning, eccentric motion, strain sensing, dielectric properties of liquids and density of liquids, low pressure, etc., are very low [4,16,17,18,19,20,21,22,31,32,33,34,35,36,37,38,39,40]. It is of crucial importance that these small changes of the resonant frequency are measured as accurately as possible, before they are transformed into the measured quantity.

The existing methods of measuring frequency difference by using two quartz oscillators or one quartz oscillator in a switching mode regime have high sensitivity for the frequency differences of the order of kHz and larger. The method with a modulation of two frequencies [13,29,30] of approximately 4 MHz uses a low pass filter to filter the frequency difference, which is then measured by a programmable counter (e.g., HM 8123, Rohde & Schwarz Company, Munich, Germany) [41]. To obtain the highest possible sensitivity, we need temperature compensation of the quartz crystals (difference of temperature–frequency characteristics between both quartz crystals is lower than 0.01 ppm), compensation of parasitic impedance effects in the oscillator circuits, compensation of aging of oscillator elements and both crystals must have similar resonant conditions in the MHz region. In this case, the error in the frequency measurement is of the order of ±0.01 Hz, and at a frequency difference of 10 kHz, the relative error is of the order of $10^{-6}$. With a reducing frequency difference, the relative error increases and it is $10^{-2}$ for 1 Hz of difference. Requirements for compensations are avoided if a method with a switching quartz sensor is used [15]. In this method, two impedances (capacitance or inductance), which are in series with the quartz crystal. were switched in a positive feedback loop within one quartz oscillator. Using this method, we obtained two different frequencies (close to the resonant frequency, e.g., 5 MHz) separately, which were affected by the same temperature drift. An additional reference oscillator was used to transform the frequencies in the range of kHz. When these two frequencies were subtracted, the effect of the temperature dependence of the reference frequency is eliminated as well. The two frequencies were then measured by a programmable counter with the same resolution as the modulation method.

In this paper we proposed a setup that enables very accurate measurements of low frequency differences, of the order of Hz, that is, in the region where the above-mentioned methods fail [41,42]. By being able to measure frequency differences of the order of 1 Hz (as opposed to kHz) means, we can detect changes in the externally measured physical quantities that are $10^{3}$-times lower. The frequency difference is measured between two 4 MHz quartz oscillators, which together act as a sensor. The method employs a new external gate signal former mode approach to measure transitions through zero within one beat period. By using this method, the relative error of the measured frequency difference is reduced by several orders of magnitude compared to the above-mentioned methods and, rather contra intuitively, the error reduces when the frequency difference is lowered. To apply the method, the same conditions must be met as for the modulation method: temperature compensation of the quartz crystals, compensation of parasitic impedance effects in the oscillator circuits and compensation of aging of oscillator elements. However, for the method to be successful, both crystals must have equal resonant conditions in the MHz region and their resonant frequencies should not differ by more than few Hz. Because the resonant frequencies are so close, we can also achieve a better temperature compensation with the difference of temperature frequency characteristic between both quartz crystals being lower than 0.001 ppm.

The paper is structured as follows. In the next section we give a theoretical background to relate the frequency difference with the number of transitions through zero within one beat period and discuss the relative error of the obtained frequency difference. Then we present the experimental setup to measure the frequency difference by counting the transitions through zero within one beat period by using an external gate signal former and counter. Finally, we present and discuss experimental results.

## 2. Obtaining the Frequency Difference from One Beat Period

**Figure 1:**
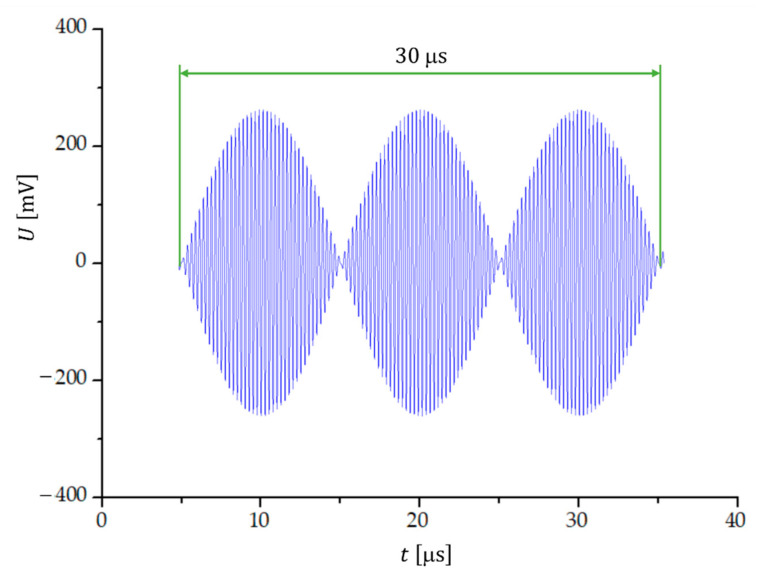
Formation of a beat signal when two signals with frequencies $f_{1}=3.95$ MHz and $f_{2}=4.05$ MHz are summed. The beat period is 10 μs.

When two sinusoidal signals with frequencies $f_{1}$ and $f_{2}$ are summed, a characteristic beat pattern is observed (Figure 1) if the frequency difference $\Delta f=f_{2}-f_{1}$ is much smaller than the two frequencies. The beat frequency is simply Δf, thus the period of one beat ($T_{1b}$) is $T_{1b}=1/\Delta f$. The frequency of the summed signal is $\left(f_{1}+f_{2}\right)/2$, so the period of one oscillation ($T_{1o}$) is $T_{1o}=2/\left(f_{1}+f_{2}\right)$. Within one beat period there are $T_{1b}/T_{1o}$ cycles, which means that there twice as many transitions through zero ($N_{zero})$:

(1) $$\begin{array}{c} N_{zero}=\frac{f_{1}+f_{2}}{\Delta f}, \end{array}$$

from where we calculate the frequency difference:

(2) $$\begin{array}{c} \Delta f=\frac{f_{1}+f_{2}}{N_{zero}}. \end{array}$$

The relative error of the measured frequency difference ($\Delta f_{err}$) depends on the relative error of the counted number of transitions through zero and the relative error of the measured sum of the frequencies:

(3) $$\begin{array}{c} \Delta f_{err}=\frac{\Delta N_{zero}}{N_{zero}}+\frac{\Delta \left(f_{1}+f_{2}\right)}{f_{1}+f_{2}}. \end{array}$$

By the method proposed in this paper, we are measuring the frequency difference between two quartz oscillators, where the frequency of one oscillator is affected by some external effect, which causes a change in the equivalent circuit of one quartz crystal and in such a way affects the oscillation frequency. If we are interested in detecting very small changes, the change in, say, $f_{2}$ will also be very low. In the case of two 4 MHz quartz oscillators, the sum of two frequencies is thus $f_{1}+f_{2}=8\;\mathrm{MHz}$. If each frequency is measured with an accuracy of ±0.01 Hz (measurement by a 10-digit frequency counter), the second term on the right-hand side of Equation (3) is of the order of $10^{-9}$. As will be shown below, by a proper triggering of an arming signal, we can achieve that $\Delta N_{zero}=\pm \;1$ when two sinusoidal signals are compared. If Δf=1 Hz, the number of counted transitions through zero will be $\approx 8\cdot 10^{6}$, thus the first term on the right-hand side of Equation (3) will be of the order of $10^{-7}$. This relative error is much higher than the relative error due to the measurement of the sum of the frequencies. If the frequency difference increases, the number of the measured transitions decreases and the corresponding relative error increases. When the frequency difference is lower than 1 Hz, the second term in the right-hand side of Equation (3) should be considered as well, and eventually, at very low (sub-Hz) frequency differences defines the achievable accuracy of the method.

The above reasoning is valid when the stability of the quartz crystal frequency is achieved, which means, as already explained in the introduction, that the quartz crystals are temperature-compensated, parasitic impedance effects in the oscillator circuits are compensated, aging of the oscillator elements is compensated, both crystals must have equal resonant conditions in the MHz region and their resonant frequencies should not differ by more than a few Hz.

## 3. Experimental Setup

**Figure 2:**
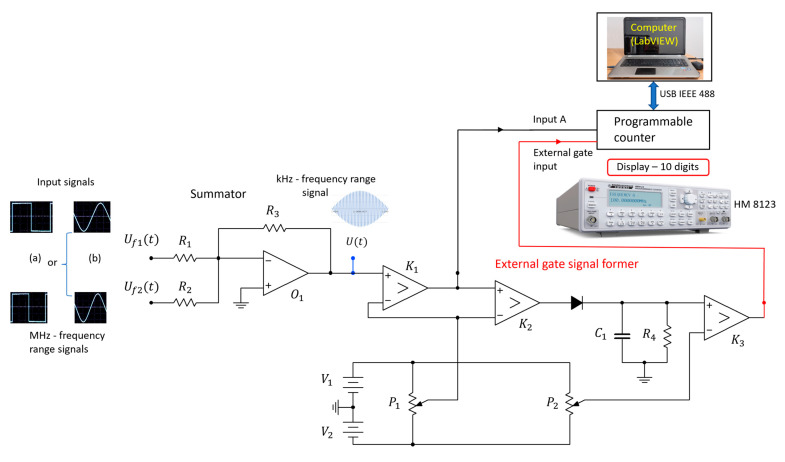
Experimental setup to count the number of transitions through zero within one beat period. Input signals are of (**a**) rectangular or (**b**) sinusoidal form.

The experimental setup is shown in Figure 2. We developed a method that counts the number of transitions through zero within one beat period when frequencies $f_{1}$ and $f_{2}$ of two signals $U_{f1}\left(t\right)$ and $U_{f2}\left(t\right)$, respectively, obtained from two quartz oscillators are temperature-compensated, both quartz crystals in oscillators have the same resonance conditions, parasitic effects are the same for both signals, the elements in both oscillators are of the same quality and both signals have the same amplitude [12,14,43]. Sensors based on two coupled quartz oscillators were developed previously and are described in Refs. [13,29,30]. We use a summator with an operational amplifier $O_{1}$ for summing up the two signals. The input signals can be of a sinusoidal or rectangular shape. The output voltage on the summator is $U\left(t\right)=-\left(U_{f1}\left(t\right)+U_{f2}\left(t\right)\right)$ for alternating signals, providing that all three resistors (see Figure 2) have the same resistance: $R_{1}=R_{2}=R_{3}$. At the output of the summator we obtain a characteristic beat signal. The frequency of the beat signal depends on the frequency difference of the measured signals. The number of oscillations within one beat period also depends on the frequency difference between the signals $U_{f1}\left(t\right)$ and $U_{f2}\left(t\right)$ (see Equation (1)). For an accurate measurement of the number of these oscillations and for triggering the measurement we use a programmable counter HM 8123 in the external gate mode of operation, which is triggered by an external gate signal former. The output signal from the summator is connected to the input A (Channel A) of a programmable counter, where it is filtered by a low pass filter (LPF)–a chosen function in the counter–to remove possible higher frequency disturbances.

**Figure 3:**
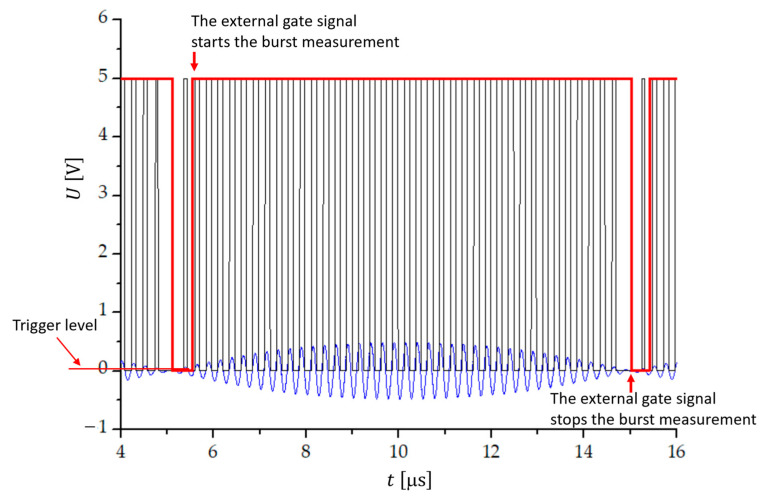
Temporal sequence of signals when the number of transitions through zero within one beat period is counted with the help of an external gate signal. The measuring time is synchronized with the first transition through zero of one beat period and trigger level of 0.5 mV.

By using the external gate mode operation (Figure 2) of the programmable counter, the counting of the transitions through zero can be defined with a high accuracy [41]. The external gate signal former consists of a comparator $K_{1}$, by which negative amplitudes of the beat signal are removed. At the exit of the comparator $K_{1}$, we obtain rectangular pulses with an amplitude of 5 V (black line in Figure 2). The comparator $K_{1}$ switching level is in the range 0.5–20 mV. The signal then goes through an envelope detector (diode, $C_{1},\;R_{4})$, where the envelope of one beat period is filtered out. In the comparator $K_{3}$, the switching level from low to high at the beginning of the beat and from high to low at its end is set to 2.8 V. The output of the comparator $K_{3}$ is a rectangular signal (shown red in Figure 3), which presents the external gate signal that overrides the set measurement time of the counter. The external gate function of the counter allows for a full control of the start and stop of the measurement. When EXT GATE is selected by a program, run in the LabView environment, and the control input signal is low, the counter makes all the necessary preparation for the measurement. With the high level of the gate signal, the measurement starts when the input signal triggers after a synchronization delay. The measurement stops on the first trigger after the external gate signal changes from high to low.

## 4. Results and Discussion

**Figure 4:**
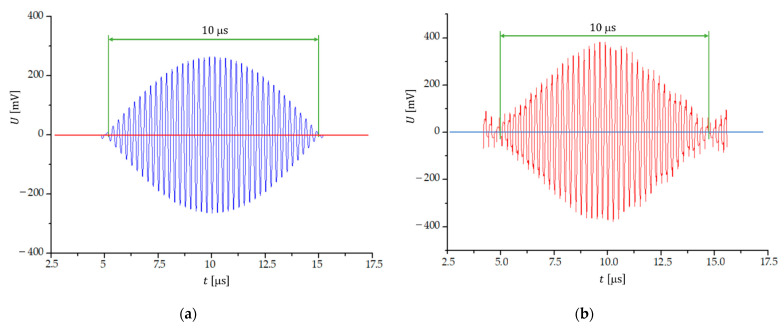
One beat period of the beat signal, when the frequency difference of two (**a**) sinusoidal and (**b**) rectangular signals is 100 kHz; $f_{1}=3.95$ MHz and $f_{2}=4.05$ MHz.

Figure 4a shows the shape of the beat signal if the input signals are sinusoidal and of equal amplitudes and the frequency difference is 100 kHz. The number of transitions through zero is 78±1. Transitions through zero are smooth and with a low noise signal. If the input signals are of a rectangular shape, we obtain a beat signal with distorted peaks (Figure 4b), which makes it difficult to determine the beginning and end of the signal and presents a significant problem for the triggering of the arming signal. As a result, the error in counting the number of transitions through zero increases to approximately ±3.

**Figure 5:**
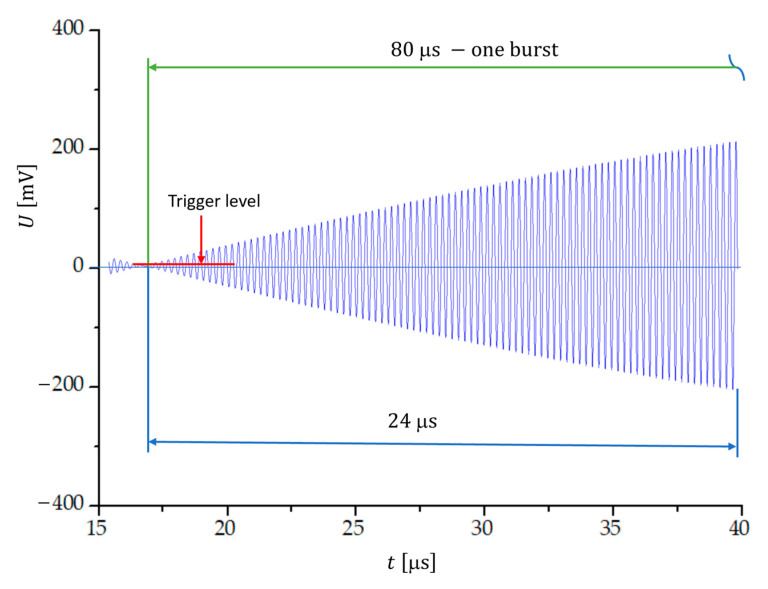
The first 24 μs of a beat signal formed by two sinusoidal signals with a frequency difference of 12.5 kHz; $f_{1}=3.99375$ MHz and $f_{2}=4.00625$ MHz. The trigger level is at 0.5 mV.

Figure 5 shows the first 24 μs of a 80 μs long beat signal obtained for two sinusoidal signals with a frequency difference 12.5 kHz at frequencies $f_{1}=3.99375$ MHz and $f_{2}=4.00625$ MHz. The increasing amplitude of the sinusoidal beat signal enables an accurate triggering of the measurement with respect to the threshold voltage. The number of transitions through zero within one beat period is 640±1.

**Figure 6:**
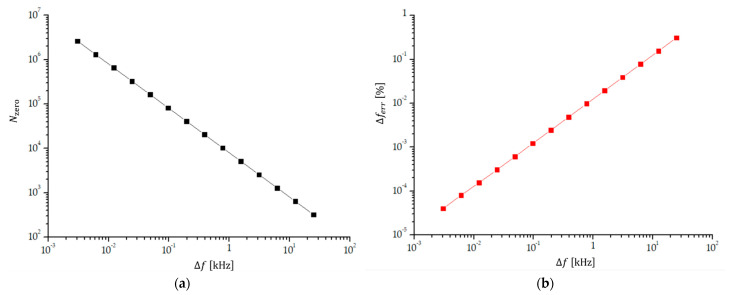
(**a**) The number of transitions through zero $\left(N_{zero}\right)$; and (**b**) relative experimental error of the measured frequency difference ($\Delta f_{err}=1/N_{zero}$) as a function of frequency difference Δf. The signal frequencies at Δf=25 kHz are $f_{1}=3.9875$ MHz and $f_{2}=4.0125$ MHz and $N_{zero}=320\pm 1$. At Δf=97.65625 Hz and $f_{1}=3.999951171875$ MHz and $f_{2}=4.0000488288125$ MHz, $N_{zero}=81920\pm 1$. The relative error is lower than 0.05% for the frequency differences lower than 4 kHz.

The number of transitions through zero is inversely proportional to the frequency difference (Equation (1)), which means that the number of transitions through zero steeply increases when the frequency difference decreases. Figure 6a shows the increase in the number of transitions through zero when the frequency difference reduces from 25 kHz to 6.103515 Hz and the frequency of the input signals is approximately 4 MHz. Because $N_{zero}\propto \Delta f^{-1}$, the log-log diagram is linear. Figure 6b shows the reduction in the relative experimental error of the measured frequency difference, $\Delta f_{err}$, which is the same as the relative error of the counted number of transitions through zero within one beat period, $1/N_{zero}$, assuming that the experimental error in counting the transitions through zero is always ±1. Because $\Delta f_{err}=N_{zero}^{-1}$ and $N_{zero}\propto \Delta f^{-1}$, the dependence of $\Delta f_{err}$ on Δf is linear, but we show the dependence on the log-log diagram to make the reduction in experimental error with reduced frequency difference clearer.

**Figure 7:**
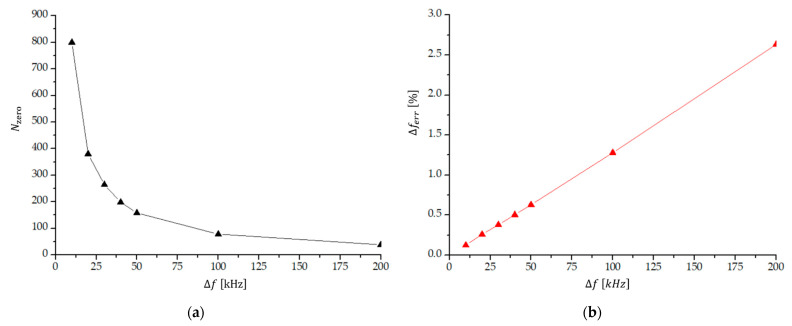
(**a**) The number of transitions through zero ($N_{zero}$) within one beat period; and (**b**) the corresponding relative experimental error of the measured frequency difference ($\Delta f_{err}=1/N_{zero}$) as a function of a frequency difference Δf in the region 12 kHz to 200 kHz.

Figure 7a shows the number of transitions through zero within one beat for larger frequency differences (12 kHz to 200 kHz). The relative experimental error of the counted number of transitions through zero within one beat period (and thus the relative error of the measured frequency difference) is shown in Figure 7b. We see that the method of counting transitions through zero within one beat period in this frequency range is not a useful experimental technique, because the experimental error is rather high. At frequencies larger than 40 kHz, the measuring error is already larger than 0.5%.

**Figure 8:**
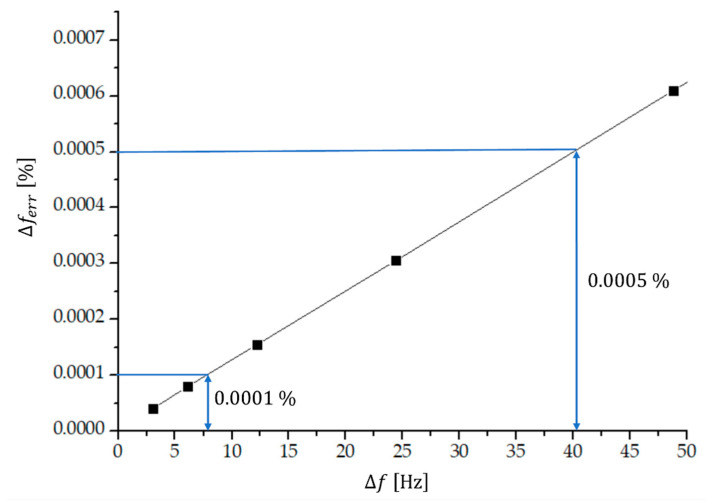
The relative experimental error of the measured frequency difference as a function of the frequency difference Δf being lower than 50 Hz. See also Table 1.

The real value of the proposed experimental technique is in measuring very low frequency differences because the measuring error decreases with a decreasing frequency difference as explained in Section 2. Some frequency differences and the corresponding number of transitions through zero including the experimental error are given in Table 1, for the case of sinusoidal input signals $U_{f1}\left(t\right)$ and $U_{f2}\left(t\right)$. We see that the experimental error becomes lower than 0.01% if the frequency differences are lower than approximately 780 Hz (at $f_{1}$ and $f_{2}$ being approximately 4 MHz) and it reduces below 0.001% when the frequency difference is lower than approximately 50 Hz. The relative experimental error as a function of frequency difference for differences lower than 50 Hz is shown also on Figure 8. We see that the relative experimental error of the measured frequency difference is 0.0005% for frequency difference Δf. approximately 40 Hz and it is 0.0001% at approximately 8 Hz.

Table 1 and Figure 8 show an excellent accuracy of the proposed method at low frequency differences, provided that the environmental temperature is stabilized and we use a temperature pair of quartz crystals (4 MHz) with a temperature difference of two temperature–frequency characteristics of the AT cut crystals, defined by the quartz crystal producers [43], being lower than 0.001 ppm. As explained at the end of Section 2, the lower limit of the relative error of the measured frequency difference is of the order of $10^{-9}$, defined by the relative error of the measured frequencies $f_{1}$ and $f_{2}$. This limit is reached for the frequency differences of the order of 0.1 Hz.

## 5. Conclusions

We have demonstrated an experimental setup to measure small frequency differences in dual-frequency mode 4 MHz quartz sensors by measuring the number of transitions through zero within one beat period of the beat signal. The major advantage of the method is that the relative experimental error reduces when the measured frequency difference reduces, thus very small frequency changes (of the order of Hz or even less) can be measured with an extremely small experimental error. The major results are summed in Table 1 and Figure 8, where we see that the relative experimental error for measuring the frequency difference of the order of 1 Hz is of the order of $10^{-7}$, which is five orders of magnitude lower compared to the method with a modulation of two frequencies or a switching mode method. This is several orders of magnitude lower compared to other methods. The presented method opens new applications in physics, chemistry, pharmacy, mechatronics, biosensorics and industrial applications, where high quality measurements are required and where changes in the measuring quantities are very small.

This proposed method for measuring very small frequency differences is important for sensors, where we want to ensure as equal as possible resonant conditions for operation of two quartz crystals in oscillators, the model parameters of which are manipulated in the equivalent circuit in the fF or aF region. To ensure that conditions are as equal as possible, the effects of temperature, humidity, external capacitances and inductances which affect the equivalent circuit of the oscillators must be addressed, as well as the parasitic impedances, which affect crystal oscillations.

The experimental approach, which uses counting the transitions through zero within one beat period (Figure 2), requires that both frequency signals have undergone the compensation of the quartz crystal temperature characteristic, parasitic impedances, and the effect of aging of the oscillator circuit, and that both crystals have the same resonant conditions—equal capacitive or inductive influence on the equivalent circuits of the crystals. In addition, the counter requires an external gate signal former. This signal overrides the set measurement time of the counter and limits the measuring time to one beat period.

To sum up, the method of counting the number of transitions through zero within one beat period enables a new approach to measure the frequency difference by which the experimental error reduces if the measured frequency difference reduces. This enables measurements of very small changes in capacitance, induced by some small change of an external physical quantity that we want to measure, with an accuracy that is five orders of magnitude higher than for the method with a modulation of two frequencies or a switching mode method, which both have the relative error of the order of $10^{-2}$ if the frequency difference is of the order of 1 kHz. Because the measurement time is defined by one beat period, the method enables the development of very fast sensors of very small changes of physical quantities.

## Figures and Tables

**Table 1:** The measured frequencies ($f_{1}$ and $f_{2}$), their difference Δf, number of the counted transitions through zero ($N_{zero}$) within one beat period and the corresponding experimental error of the measured frequency difference ($\Delta f_{err}=1/N_{zero}$) assuming that $N_{zero}$ is measured with experimental error ±1.

	$\Delta f\;\left(kHz\right)$	$f_{1}\;\left(MHz\right)$	$f_{2}\;\left(MHz\right)$	$N_{zero}$	$\Delta f_{err}$ **(%)**
1	25.000000000	3.987500000000	4.012500000000	320	0.31250
2	12.500000000	3.993750000000	4.006250000000	640	0.15625
3	6.250000000	3.996875000000	4.003125000000	1280	0.07813
4	3.125000000	3.998437500000	4.001562500000	2560	0.03906
5	1.562500000	3.999218750000	4.000781250000	5120	0.01953
6	0.781250000	3.999609375000	4.000390625000	10240	0.00976
7	0.390625000	3.999804687500	4.000195312500	20480	0.00488
8	0.195312500	3.999902343750	4.000097656250	40960	0.00244
9	0.097656250	3.999951171875	4.000048828125	81920	0.00122
10	0.048828125	3.999975585937	4.000024414063	163840	0.00061
11	0.024414063	3.999987792969	4.000012207031	327680	0.00031
12	0.012207030	3.999993896485	4.000006103515	655360	0.00016
13	0.006103515	3.999996948243	4.000003051757	1310720	0.00008
14	0.003051756	3.999998474122	4.000001525878	2621440	0.00004

## Data Availability

Not applicable.
